# Mining of linear B cell epitopes of SARS-CoV-2 ORF8 protein from COVID-19 patients

**DOI:** 10.1080/22221751.2021.1931465

**Published:** 2021-06-03

**Authors:** Xiaohui Wang, Joy-Yan Lam, Linlei Chen, Shannon Wing-Ngor Au, Kelvin K. W. To, Kwok-Yung Yuen, Kin-Hang Kok

**Affiliations:** a Department of Microbiology, Li Ka Shing Faculty of Medicine, The University of Hong Kong, Hong Kong SAR, People’s Republic of China; b School of Life Sciences, The Chinese University of Hong Kong, Hong Kong SAR, People’s Republic of China; c State Key Laboratory for Emerging Infectious Diseases, The University of Hong Kong, Hong Kong SAR, People’s Republic of China; d Carol Yu Centre for Infection, The University of Hong Kong, Hong Kong SAR, People’s Republic of China; e AIDS Institute, Li Ka Shing Faculty of Medicine, The University of Hong Kong, Hong Kong SAR, People’s Republic of China

**Keywords:** SARS-CoV-2, ORF8, COVID-19, peptide, epitope, antibody

## Abstract

Given the on-going SARS-CoV-2 pandemic, identification of immunogenic targets against the viral protein will provide crucial advances towards the development of sensitive diagnostic tools and vaccination strategies. Our previous study has found that ORF8 protein of SARS-CoV-2 is highly immunogenic and shows high sensitivity in identifying COVID-19 disease. In this study, by employing overlapping linear peptides, we characterized the IgG immunodominant regions on SARS-CoV-2 ORF8 protein that are seropositive in the sera from SARS-CoV-2-infected patients. The major immunogenic epitopes are localized at (1) N-termini alpha helix, (2) the resides spanning beta 2 and 3 sheets, and (3) the loop between beta 4 and 5 sheets. Additionally, hamster model infected by SARS-CoV-2 further validates the seropositivity of the linear epitopes *in vivo*, demonstrating a potential application of the linear peptide-based immunization strategy. Taken together, identification and validation of these B-cell linear epitopes will provide insights into the design of serological diagnostics and peptide-based vaccination approach against this pandemic virus of high priority.

## Introduction

Novel Severe Acute Respiration Syndrome-Coronavirus-2 (SARS-CoV-2) continues to be a significant global public health challenge. The major manifestations of the SARS-CoV-2 infection-caused novel coronavirus disease 2019 (COVID-19) are the predominant respiratory symptoms, and also systemic inflammation which may lead to multi-organ dysfunction, including acute respiratory distress syndrome, cardiovascular disorders, disseminated intravascular coagulation and neurological symptoms [1–4]. The presence of many asymptomatic patients with high viral load may contribute to its high transmissibility. In the race to develop and practise COVID-19 vaccine, we are more eager to understand immunobiology underlying vaccine response than before. Therefore, the knowledge on the immune response to SARS-CoV-2 is of utmost importance.

Unlike other human highly pathogenic coronaviruses, SARS-CoV-2 genome contains a unique open reading frame (ORF) 8 gene [5]. Our analysis on the protein sequence showed that SARS-CoV-2 ORF8 is a novel protein which is different from those in other coronaviruses. ORF8 is absent in all human pathogenic coronaviruses except the recent SARS-CoV-2, but present in some bat coronaviruses. We found that SARS-CoV-2 ORF8 is a putative secreted protein with an N-terminal signal peptide [5]. Further validation found that ORF8 protein is secreted into the sera of COVID-19 patients and transfected cell lines [6]. We previously found that SARS-CoV-2 proteins, such as ORF6, function as interferon antagonists, but ORF8 does not interfere interferon signalling pathway as potently as ORF6 [7]. A recent study suggested that ORF8 mediates immune evasion by downregulating MHC-I molecules [8], which likely exacerbates the crippled B and T cell responses in COVID-19 patients [9–13]. Although *in vitro* studies suggested that mutations or deletions in ORF8 (Δ382 variant) are not associated with SARS-CoV-2 replication [14], milder clinical features are found in Δ382-infected COVID-19 patients [15]. In addition, recently emerged UK variant 20I/501Y.V1 (also known as B.1.1.7 lineage) contains a substitution that creates an early stop codon of ORF8 at position 27 [16]. Notably, the mass spectrometry-based interactome analysis identified 15 out of 47 human proteins that interact with ORF8 are known drug targets [17]. Taken together, it is likely that ORF8 plays a biological role in the pathogenesis of SARS-CoV-2.

Almost all the current COVID-19 vaccines under development or testing are focused on spike protein. Recent studies characterized immunogenic epitopes of spike protein using linear peptides, providing insight into utilizing peptides as diagnostic tools and vaccination candidates [18,19]. Despite its undefined role *in vivo*, we and others previously found SARS-CoV-2 ORF8 is a unique protein that is highly immunogenic in COVID-19 patients at early and late stages of disease [6,20]. ORF8 epitopes have been identified on human and mouse T cells using synthesized peptides and *in silico* prediction, whereas the B cell epitopes remain unidentified [21–23]. We designed a peptide pool that covers the whole length of ORF8 and assessed the antibody profiles of COVID-19 patients. Three groups of immunodominant linear B cell epitopes, on the ORF8 protein of SARS-CoV-2, were identified in COVID-19 patients and SARS-CoV-2-infected hamsters. These epitopes may be potentially used in the design of serological assays for epidemiological or vaccine efficiency assessments.

## Materials and methods

### Ethics statement

Written informed consent was obtained from participants in accordance with the tenets of the Declaration of Helsinki. COVID-19 patients with respiratory samples positive reverse-transcription quantitative PCR (RT-qPCR) for SARS-CoV-2, after admission into Queen Mary Hospital in Hong Kong during January and July 2020, were included in this study, as we previously reported [6]. In-house reverse-transcription quantitative PCR (RT-qPCR), targeting the SARS-CoV-2 RNA-dependent-RNA-polymerase-helicase gene region, was performed as we previously described [24,25]. In Hong Kong, patients were tested for SARS-CoV-2 based on clinical and epidemiological criteria as outlined and updated by the Hospital Authority. Final confirmation of initially positive specimens, using nasopharyngeal or sputum specimens, was done at the Public Health Laboratory Centre of Hong Kong. The negative control sera were collected from Hong Kong blood donors in October 2019 (before the emergence of COVID-19). This study was approved by the Institutional Review Board of the University of Hong Kong/Hospital Authority Hong Kong West Cluster (UW 13-372). Since archived specimens were used, written informed consent was waived.

### Linear peptide library

The sequences, used for the design of linear peptides of the ORF8 protein of SARS-CoV-2, are under NCBI Reference Sequence: YP_009724396.1. Preliminary epitope screening was used with a library of peptides (GenScript) consisting of 15-mer peptides overlapping by 10 amino acids spanning ORF8 sequence (Table S1). The peptides were generated using solid phase synthesis methods and the quality of the products was monitored by mass spectrometry. Lyophilized individual peptides were dissolved in DMSO (Sigma-Aldrich) to obtain a stock solution.

### Peptide-based ELISA

Peptide ELISA was performed in a similar manner to a previously established ORF8 ELISA assay [6]. In brief, all peptides, at a final concentration of 5 μg/mL with 50 μL per well in pH 7.6 phosphate buffered saline (PBS) buffer, were coated on Nunc Maxisorp flat-bottom 96-well plates (Thermo Fisher Scientific) overnight at 4°C and then blocked with 0.05% PBST (0.05% v/v Tween-20, Sigma-Aldrich, in PBS, Gibco) containing 0.5% w/v. Gelatin (Sigma-Aldrich) and 0.5% w/v bovine serum albumin (BSA; Sigma-Aldrich). Heat-inactivated patient serum samples were added at 1:100 dilution in PBS containing 0.5% BSA. Anti-Human IgG (*γ*-chain specific), F(ab′)2 fragment−Horseradish peroxidase-conjugated antibody produced in goat (SIGMA, #A2290-1ML) prepared in PBS containing 0.5% BSA, was used for the detection of peptide-bound antibodies. In total, 50 μL of TMB substrate (Thermo Fisher, #34028) was used for a 20-min development and was stopped by the addition of 20 μL of 0.16 M sulphuric acid prepared from 95% to 97% Sulphuric Acid stock solution (Merck), prior to absorbance measurements. Absorbance was measured at 450 nm using a Varioskan LUX Multimode Microplate Reader (Thermo Fisher Scientific). The cut-off for seropositivity was set as the mean value of 100 control serum samples plus two times of standard deviation.

### Recombinant SARS-CoV-2 ORF8 protein production and purification

The Expi293 expression system (Thermo Fisher Scientific) was used for mammalian recombinant ORF8 protein production. Expi293F cells were transfected with pCAGEN-ORF8-His using Expifectamine 293 (Thermo Fisher Scientific) according to the manufacturer’s instructions. After 5–6 days’ post-transfection, serum-free medium, carrying secreted ORF8 protein, was harvested and purified by anti-his affinity resin (GenScript, China). Recombinant His-ORF8 protein was eluted using low pH amine-based elution buffer (IgG Elution Buffer, ThermoFisher), which is immediately neutralized with 1M Tris-Cl, pH 8.8.

### Virus and biosafety

SARS-CoV-2 was isolated from the nasopharyngeal aspirate specimen of a laboratory-confirmed COVID-19 patient in Hong Kong [26]. The plaque-purified virus isolate was amplified by one additional passage in VeroE6 cells to make working stocks of the virus, as described previously [26]. All experiments involving live SARS-CoV-2 followed the approved standard operating procedures of the HKU Biosafety Level-3 facility.

### Illustration of corresponding peptide on the full-length ORF8 protein

The full-length structure (amino acid position 1–121) was modified from the published mature ORF8 protein structure (amino acid 18–121; PDB: 7JTL). The N-terminus of ORF8 was predicted using a comparative modelling by the Robetta protein structure prediction server. The full-length ORF8 (N-terminus 1–17 and mature 18–121) were visualized, coloured, and integrated using the PyMOL (Delano Scientific LLC) software.

### Hamster infection

Approval was obtained from the HKU Committee on the Use of Live Animals in Teaching and Research. Male and female Syrian hamsters, aged 6–10 weeks old, were obtained from HKU Laboratory Animal Unit. The animals were kept in Biosafety Level-2 housing and given access to standard pellet feed and water *ad libitum* until virus challenge in our Biosafety Level-3 animal facility. PBS was used to dilute virus stocks to the desired concentration, and inocula were back-titrated to verify the dose given. Dulbecco’s Modified Eagle Medium (DMEM), containing 10^5^ plaque-forming units in 100 µL of SARS-CoV-2, was intranasally inoculated under intraperitoneal ketamine (200 mg/kg) and xylazine (10 mg/kg) anaesthesia. Mock-infected animals were challenged with 100 µL of PBS. Animals were monitored twice daily for clinical signs of disease, as described previously [27]. Animals were sacrificed at 14 dpi and sera were collected.

### Statistics

All analyses were performed using GraphPad PRISM software. Paired or unpaired t-test was used for two group analyses and one-way ANOVA for multiple group analysis. Non-parametric data were analysed by the Mann–Whitney test. *p* values of less than 0.05 were considered statistically significant. Data in this study were indicated as mean with standard deviation.

## Results

### Characterization of immunogenic linear epitopes of SARS-CoV-2 ORF8 protein

**Figure 1.**
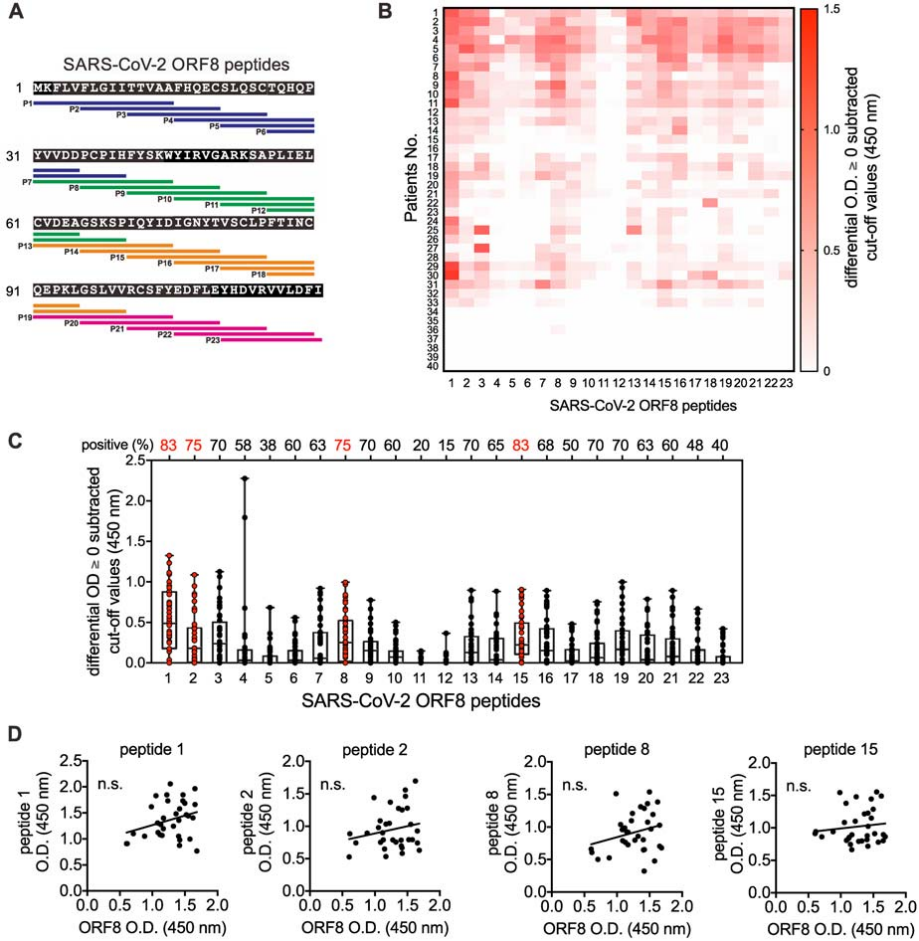
Identification of immune linear epitopes of SARS-CoV-2 ORF8 protein using the serum of COVID-19 patients. (A) Localization and sequences of the peptides on SARS-CoV-2 ORF8 protein (NCBI Reference Sequence: YP_009724396.1) as shown. (B) Sera of COVID-19 patients (*n* = 40) at 3–64 days post-symptom onse at 1:100 dilution were subjected to peptide-based ELISA using paptide covering the entire ORF8 protein in (A). The landscape of adjusted epitope-specific antibody levels in each COVID-19 patient (*n* = 40) is shown. The ELISA results as patient OD values subtracted of cut-off values are presented, negative values are plotted as zero. The cut-off for seropositivity was set as the mean value of individual control serum sample or serum pools of serum taken in October 2019 (*n* = 100) plus two times of standard deviation. (C) The seropositive rates of each peptide in COVID-19 patients (*n* = 40) in (B) were determined. (D) Associations of anti-ORF8 peptide 1, 2, 8 and 15 and anti-ORF8 recombinant protein IgG in the sera of COVID-19 patients at 13–64 days’ post-symptom onset (*n* = 33). *p* Values were calculated by Pearson correlation coefficients. Data were presented as mean ± standard deviation. Not Significant, n.s. *p* > .05.

To examine the immunodominant epitopes of ORF8 protein, we assessed linear antigenic targets from the sera of 40 COVID-19 patients at 3–69 days post-symptom onset, using a linear B-cell peptide library spanning the entire ORF8 protein of SARS-CoV-2. Each peptide was 15 residues in length with a 10-residue overlap (Figure 1(A)). Sera from healthy donors collected in 2019 were used as controls. As those COVID-19 patients have been confirmed to be seropositive to ORF8 protein by enzyme-linked immunosorbent assay (ELISA) assay (data not shown), the immunodominant sites in terms of the positive rate and the percentage of the sera may be revealed by the positive reactions to the epitopes using peptide-based ELISA. The epitope mapping showed that 85% (34/40) of the examined patients are positive to specific peptides to a certain extent (Figure 1(B) and Table S2), with 18 linear sites on the ORF8 protein having a positive rate of ≥50% among all examined patients (Figure 1(C)). Within the peptide pool, peptides 1, 2, 8 and 15 were recognized in ≥75% COVID-19 patients but not healthy donors (Figure 1(C)). However, the OD values of the selected peptides did not show a proportional correlation with OD values of the recombinant ORF8 protein-based ELISA assay (Figure 1(D)). Taken together, these results illustrated the location of the linear epitopes in ORF8 protein, in which the N-termini alpha helix exhibits high immunogenicity.

### ORF8 linear epitopes-specific antibodies can be detected in SARS-CoV-2-infected hamsters

**Figure 2.**
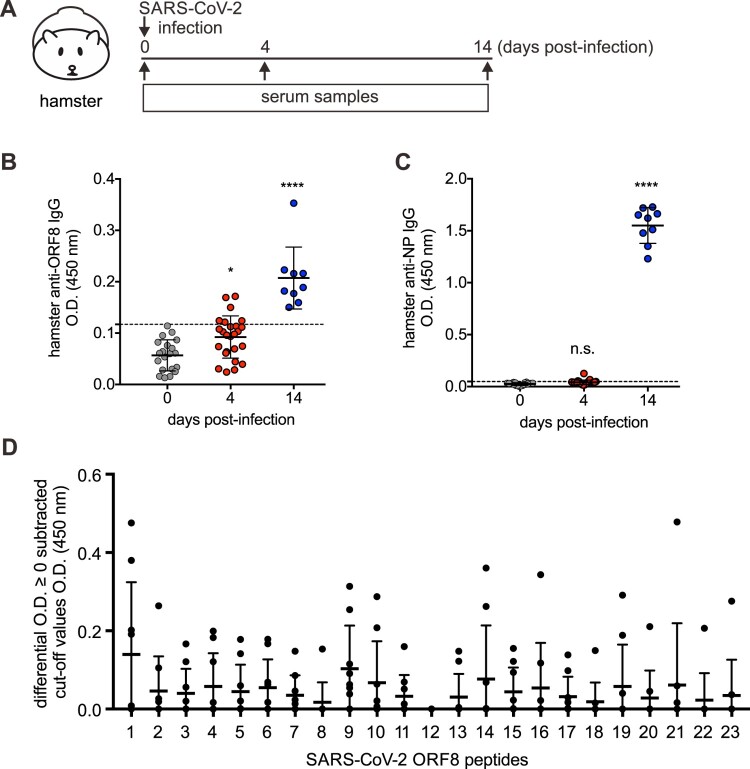
Epitope immunodominance of SARS-CoV-2 ORF8 protein in SARS-CoV-2-infected hamsters. (A–C) Hamsters were infected with SARS-CoV-2 virus (A). Antibodies agasint ORF8 protein (B) and N protein (C) in the sera of SARS-CoV-2-challenged hamsters at 0 (*n* = 20), 4 (*n* = 25), and 14 dpi (n=9). (D) Sera of SARS-CoV-2-infected hamsters at 14 dpi (*n* = 9) at 1:100 dilution were subjected to peptide-based IgG ELISA using peptide pools covering the entire ORF8 protein of SARS-CoV-2. Sera of naïve hamsters (*n* = 20) were assessed in parallel. OD values subtracted of cut-off values presented with negative values are plotted as zero. The cut-off for seropositivity was set as the mean value of control serum samples plus two times of standard deviation. The dotted lines represent cut-off values. Data were presented as mean ± standard deviation. *, *p* < .05; **, *p* < .01; ***, *p* < .001; ****, *p* < .0001.

To further examine the immunogenicity of SARS-CoV-2 ORF8, we utilized experimental hamster model with SARS-CoV-2 infection which has been successfully established in a previous study [27] (Figure 2(A)). Sera from SARS-CoV-2-infected hamsters were collected and subjected to our in-house anti-ORF8 and SARS-CoV-2 nucleoprotein (N) ELISA assays [6,25]. Consistent with our previous finding that seroconversion of anti-ORF8 IgG is much earlier than anti-N in COVID-19 patients, anti-ORF8 IgG was detected at earlier time-point than anti-N IgG in SARS-CoV-2-infected hamsters (Figure 2(B,C)). Similarly, a significant seropositivity against multiple peptides was detected in sera from infected hamsters, with relative higher reading against peptide 1 (Figure 2(D)). Taken together, the results from animal models demonstrate that the linear peptides are potentially applicable to animal studies.

### Localization of predicted immunogenic epitopes on ORF8 protein

**Figure 3.**
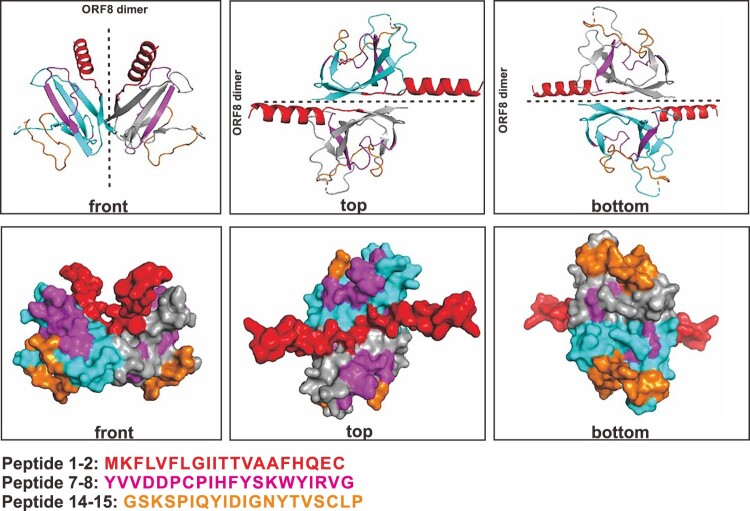
Mapping of linear peptides on the modified ORF8 protein structure. Regions of representative immunogenic epitopes on full-length ORF8 dimer are shown as red (peptide 1–2), pink (peptide 7–8) and orange (peptide 14–15). The full-length structure (amino acid position 1–121) was modified from the published mature ORF8 protein structure (amino acid 18–121; PDB: 7JTL). The monomers are coloured in cyan and grey.

Recent structural study revealed the SARS-CoV-2 ORF8 homodimerizes in the presence of one inter-molecular disulphide linkage [28]. The dimer structure was resolved using the ORF8 protein ranging from amino acid position 18–121 (PDB: 7JTL). We recently predicted the structure of ORF8 that includes the N-termini alpha helix (amino acid position 1–17) [6]. To demonstrate the localization of predicted immunogenic epitopes on ORF8 protein, we simulated and integrated the N-termini alpha helix into the published ORF8 dimer structure (PDB: 7JTL). The major sets of the immunogenic epitopes are localized at the N-termini alpha helix (epitopes 1–2), resides spanning β2 and β3 sheets (epitopes 7–8), and the loop between β4 and β5 (epitopes 14–15) (Figure 3). Notably, the resides spanning from β5 to β7 sheets (epitopes 18–22) localize in close proximity to the dimerization interface that is formed unique to SARS-CoV-2 ORF8 monomer [28]. Taken together, these results illustrated the linear B cell epitopes against ORF8 of SARS-CoV-2, demonstrating a potential in utilizing peptides to detect SARS-CoV-2-exposed subjects and as immunogens.

## Discussion

We and others have reported that ORF8 protein is highly immunogenic in COVID-19 patients [6,20]. In this study, we further identified immunodominant linear B-cell epitopes on the SARS-CoV-2 ORF8 protein with sera from COVID-19 patients at early and late stages of SARS-CoV-2 infection. This approach uses easily synthesized linear peptides, which will greatly benefit the scientific community in allowing rapid and safer assessments of ORF8 antibody titres in patient blood and potential application of monoclonal antibodies. Animal model using SARS-CoV-2-infected hamsters functionally validated the immunogenicity of the identified epitopes in vivo. Future studies will be needed to fully understand the immunogenicity of linear peptides in experimental animal models.

The activation of the immune system in response to SARS-CoV-2 infection and the clinical sequela are complex, with a general crippled immune response found in COVID-19 patients [9–13]. Our previous work revealed that the titres of anti-N IgG, IgM and IgA antibodies in COVID-19 patients increase over time following infection [25]. However, waning of spike-specific neutralizing antibodies was found in convalescent COVID-19 patients [9,10]. These instances of no or low antibody responses against spike and N corroborated with the impaired generation of memory B cells, germinal centre formation and circulating follicular helper T cells, with the latter two being conventionally critical for the formation and maintenance of long-lasting memory formation [11–13]. These instances of no or low antibody responses against N and spike protein may lead to an underestimation of early or asymptomatic infections and threaten the success of a potential vaccine that targets the spike protein alone. Therefore, the identification of additional viral targets, such as ORF8, with high immunogenicity and specificity, is urgently needed for timely sero-surveillance and vaccine development.

It is most likely that early immune responses are protective, but excessive responses in the later stage may instead amplify pathogenic inflammatory outcomes, in the presence of the sustained high viral loads in the lungs, by multiple hypothetical mechanisms [29,30]. Our findings may pave the way for the development of medications that could dampen such inflammatory syndromes. Currently, the majority of studies on SARS-CoV-2 vaccines have focused on spike protein and N. Whether the presence of antibodies against ORF8, which is highly immunogenic *in vivo*, can neutralize and confer protection at an early stage of infection remains unknown. Additionally, the *in vivo* neutralization of ORF8 may rescue the potential immune evasion mediated by ORF8 [8]. Recent publications also identified immunogenic epitopes of spike protein using linear peptide [18,19], and indeed, the immunogenicity of spike peptides has been validated in mice [19]. To this end, investigations on the ORF8 epitopes recognized by human B and T cell responses are of immediate relevance for assisting candidate vaccine design and facilitating evaluation of vaccine candidate immunogenicity.

SARS-CoV-2 has been recently identified from diverse mammals. The recently reported SARS-CoV-2 in farmed minks suggested the potential of a natural reservoir of SARS-CoV-2 in farmed or wild animals which may sustain the continuous evolution of the virus and result in epidemics and/or zoonoses [31]. An *in silico* analysis, conducted on all the coding gene sequences of SARS-CoV-2 strains, originating from a range of non-human mammalian species, including pangolin, bat, dog, cat, tiger, mink and mouse, found maximum genetic homology among those animal-derived SARS-CoV-2 sequences which supports the likely evolution of these strains from a common ancestor [32]. Thus, more surveillance of the SARS-CoV-2 infections in animals in terms of transmission, clinical presentation, diagnostics, and vaccine development are warranted.

An inherent limitation, when using linear peptide as diagnostics, is unable to represent the strength of antibody response against the natural ORF8 protein. This observation is similar to other linear peptide studies of SARS-CoV-2 nucleocapsid (N) protein and receptor-binding domains (RBD) of spike protein [18,19]. However, the seropositivity and disease duration-related kinetic changes in antibody titres against the linear peptide 1 of ORF8 protein, as described here, could still influence the humoral response against ORF8 of SARS-CoV-2. To test these hypotheses, a much larger cohort of COVID-19 patients with severe and mild disease that could be matched for the covariates, including age, sex, viral load and disease severity, is required. Such future studies can enhance our understanding of the immunological mechanisms underlying variable outcomes of COVID-19.

Together, these findings will guide the design and evaluation of efficient and specific serological assays against linear epitopes as well as the design of vaccine targets during this unprecedented crisis.

## Supplementary Material

Table_S2_clean.xlsx

Table_S1_clean.docx
