# Supplementary material for: Mining of linear B cell epitopes of SARS-CoV-2 ORF8 protein from COVID-19 patients
Source: Emerg Microbes Infect. 2021 Jun 3;10(1):1016–23. doi: 10.1080/22221751.2021.1931465 (PMC8186430; doi:10.1080/22221751.2021.1931465)
Supplement: Table_S1_clean.docx [file TEMI_A_1931465_SM0262.docx]

Supplementary Table 1

Table S1. Lists of all orf8 peptides used for ELISA assay.

| Peptide number | Peptide sequences |
| --- | --- |
| 1 | MKFLVFLGIITTVAAHHK |
| 2 | FLGIITTVAAFHQEC |
| 3 | TTVAAFHQECSLQSC |
| 4 | FHQECSLQSCTQHQP |
| 5 | SLQSCTQHQPYVVDD |
| 6 | TQHQPYVVDDPCPIH |
| 7 | YVVDDPCPIHFYSKW |
| 8 | PCPIHFYSKWYIRVG |
| 9 | FYSKWYIRVGARKSA |
| 10 | YIRVGARKSAPLIEL |
| 11 | ARKSAPLIELCVDEA |
| 12 | PLIELCVDEAGSKSP |
| 13 | CVDEAGSKSPIQYID |
| 14 | GSKSPIQYIDIGNYT |
| 15 | IQYIDIGNYTVSCLP |
| 16 | IGNYTVSCLPFTINC |
| 17 | VSCLPFTINCQEPKL |
| 18 | FTINCQEPKLGSLVV |
| 19 | QEPKLGSLVVRCSFY |
| 20 | GSLVVRCSFYEDFLE |
| 21 | RCSFYEDFLEYHDVR |
| 22 | EDFLEYHDVRVVLDF |
| 23 | YHDVRVVLDFI |
